# Development of a standardized patient-reported clinical questionnaire for children with spinal pain

**DOI:** 10.1186/s12874-024-02449-2

**Published:** 2025-01-04

**Authors:** Freja Gomez Overgaard, Henrik Hein Lauridsen, Mads Damkjær, Anne Reffsøe Ebbesen, Lise Hestbæk, Mikkel Brunsgaard Konner, Søren Francis Dyhrberg O’Neill, Stine Haugaard Pape, Michael Skovdal Rathleff, Christian Lund Straszek, Casper Nim

**Affiliations:** 1 Medical Spinal Research Unit, Spine Centre of Southern Denmark, University Hospital of Southern Denmark, Kolding, Denmark; 2 Department of Regional Health Research, University of Southern Denmark, Odense, Denmark; 3 Department of Chiropractic, Faculty of Medicine, Health and Human Science, Macquarie University, Sydney, Australia; 4 Department of Sports Science and Clinical Biomechanics, University of Southern Denmark, Odense, Denmark; 5 Center for Muscle and Joint Health, Department of Sports Science and Clinical Biomechanics, University of Southern Denmark, Odense, Denmark; 6 Department of Paediatrics and Adolescent Medicine, Lillebaelt Hospital, University Hospital of Southern Denmark, Kolding, Denmark; 7 The Chiropractic Knowledge Hub, Odense, Denmark; 8 Spine Centre of Southern Denmark, University Hospital of Southern Denmark, Kolding, Denmark; 9 Department of Anesthesiology and Intensive Care Medicine, Pain Center (Smertecenter Syd, OUH), University Hospital Odense, Odense, Denmark; 10 Department of Health Science and Technology, Aalborg University, Aalborg, Denmark; 11 Department of Clinical Medicine, Center for General Practice at Aalborg University, Aalborg University, Aalborg,, Denmark; 12 Department of Physical Therapy and Occupational Therapy, Aalborg University Hospital, Aalborg, Denmark; 13 Department of Physiotherapy, University College of Northern Denmark, Aalborg, Denmark; 14 Hospital Lillebelt, Sygehusvej 24, 6000 Kolding, Denmark

**Keywords:** Pediatric, Outcome Questionnaire, Comprehensive Healthcare, Musculoskeletal Pain, Questionnaire Design

## Abstract

**Background:**

Spinal pain affects up to 30% of school-age children and can interfere with various aspects of daily life, such as school attendance, physical function, and social life. Current assessment tools often rely on parental reporting which limits our understanding of how each child is affected by their pain. This study aimed to address this gap by developing MySpineData-Kids (“*MiRD-Kids”*), a tailored patient-reported questionnaire focusing on children with spinal pain in secondary care (Danish hospital setting).

**Methods:**

The process and development of MiRD-Kids followed a structured, multi-phase approach targeted children in outpatient care. The first phase involved evidence-synthesis, expert consultations, and item formulation, resulting in the first version. The second phase involved pilot testing among pediatric spinal pain patients, leading to modifications for improved clarity and relevance. The third phase involved implementation at the Pediatric outpatient track at The Spine Centre of Southern Denmark, University Hospital of Southern Denmark.

**Results:**

MiRD-Kids was based on selected items from seven questionnaires, encompassing 20 items across six domains. Pilot testing with 13 pediatric patients facilitated modifications and finalized the questionnaire. The questionnaire includes sections for parents/legal guardians and six domains for children covering pain, sleep, activities, trauma, concerns, and treatment, following the International Classification of Functioning, Disability, and Health (ICF). Implementation challenges were overcome within a 2-month period, resulting in the clinical questionnaire MiRD-Kids a comprehensive tool for assessing pediatric spinal pain in hospital outpatient settings.

**Conclusion:**

MiRD-Kids is the first comprehensive questionnaire for children with spinal pain seen in outpatient caresetting and follows the ICF approach. It can support age-specific high-quality research and comprehensive clinical assessment of children aged 12 to 17 years, potentially, contributing to efforts aimed at mitigating the long-term consequences of spinal pain.

**Supplementary Information:**

The online version contains supplementary material available at 10.1186/s12874-024-02449-2.

## Introduction

Approximately half of all children and adolescents (hereafter referred to collectively as *children*) will experience musculoskeletal pain at some point, with substantially increasing prevalence from around the age of ten [1–3]. One of the most common pain sites is the back. Up to 30% of Danish schoolchildren experience spinal pain within a one-year period [4]. Pain can have devastating effects on the individual, along with family members and friends [5], and can interfere with school attendance, social activity, physical performance, and work productivity [6–8]. Poorer outcomes are generally associated with higher age, higher pain intensity, and disability levels at initial assessment [9].

Pain experienced from the back and neck (spinal pain) constitutes one of the highest economic and societal burdens, globally for adults [10]. However, we have limited evidence about the burden of spinal pain in children [6]. Still, we do know that when children experience persistent spinal pain, they have an increased risk of also reporting spinal pain in adulthood [11, 12]. Thus, there is a need to better understand how spinal pain affects children. Despite the potential consequences that spinal pain has for children, most of the available evidence relies on parents’ accounts [13], as there are few available questionnaires aimed at children with spinal pain [6]. Understanding spinal pain's consequences for children requires well-developed, age-appropriate, patient-reported outcome measures (PROMs) [14, 15]. While tools like the PROMIS Pediatric Profiles are widely used to assess general health domains in children, they are not specifically tailored to spinal pain, and existing musculoskeletal PROMs often focus on general pain or physical function. Children, adolescents, and adults are likely to be impacted by spinal pain differently due to differences in cognitive and physical development, and social status [6, 16]. Therefore, PROMs, typically used to assess the individual burden of spinal pain for adults (e.g., housework, employment, and sexual functions), are not suitable for children [17], and the transferability across age groups is unclear.

Without proper tools and conscientious reporting, it is nearly impossible to assess or monitor the effectiveness of treatment of pediatric spinal pain [18]. A core outcome set has been developed for evaluating spinal pain in adults and is well implemented in research [17]. We lack clear indications on how to measure the burden in children [14]. While the Functional Disability Inventory (FDI) are used for care-seeking pediatric populations, it is not specifically tailored to spinal pain. Additionally, most existing pediatric measures were developed for broader musculoskeletal conditions or general health and cannot be trusted to fully capture the specific challenges faced by children with spinal pain. An example of this is physical disability, which is well-established as a construct in adults, whereas only a few versions of disability questionnaires have been developed for children [19, 20].

We aimed to develop a questionnaire with a set of standardized questionnaires and items for children with spinal pain, integrating relevant items from existing measures while addressing gaps in spinal pain-specific domains. Further, we aimed to implement this at the Spine Center of Southern Denmark, a Danish hospital setting assessing children with spinal pain, as a clinical questionnaire.

## Method

### Procedure

The development of the clinical questionnaire MySpineData-Kids (“*MiRD-Kids”*) was carried out in three phases: 1) evidence-synthesis, expert-consultation, consensus and item formulation, 2) pilot testing, and 3) implementation. The method was based on the Consensus-based Standards for the Selection of Health Measurement Instruments (COSMIN) guidelines, specifically focusing on the steps ensuring content validity [21]. We adhered to COSMIN’s recommendations for evaluating patient-reported outcome measures (PROMs) by involving both expert consultation and testing within the target population to ensure relevance and comprehensibility of the items. The COSMIN checklist for content validity was used to guide and assess each phase of the development process (Appendix 1).

**Fig. 1:**
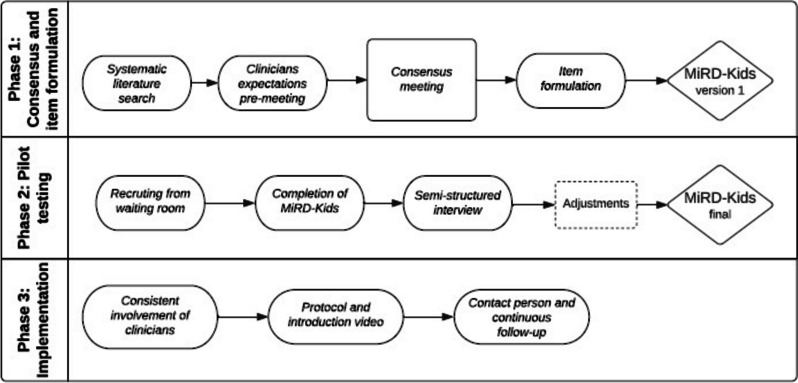
The three phases in the development of MiRD-kids

The literature search in the first phase ensured that all available questionnaires were identified and included as the basis for clinicians and experts afterwards suggesting further clinically relevant domains not covered by the available literature. This ultimately resulted in the first version of MiRD-Kids. The second phase tested MiRD-kids (version one) within a pediatric population, and this was then used to formalize the finalized clinical questionnaire. The third and final phase was the implementation of the questionnaire at the Spine Centre of Southern Denmark (Fig. 1):

### Ethics

The project was exempt from ethical approval according to Danish law by the Ethics Committee of Southern Denmark (case number: S-20232000–38) and registered with the Region of Southern Denmark (ID: 22/993).

### Study Setting

The Spine Centre of Southern Denmark is a combined medical and surgical hospital department, which serves patients with spinal pain syndromes. In addition to approximately 15.000 adults [22], more than 350 pediatric patients are seen in a separate outpatient track by a multidisciplinary team of chiropractors, medical doctors, nurses, and physiotherapists [23] that has weekly meetings to ensure age-specific treatment courses for all patients. From this cohort, we included pediatric patients (12–17 years old) to participate in phase 2: pilot testing. Phase 3 was the implementation of MiRD-Kids at the Spine Center.

### Phases

#### Phase 1: Consensus and item formulation

This phase consisted of a systematic literature search to identify relevant published questionnaires, a meeting with the clinicians of the multidisciplinary team to obtain their clinical perspective on the children and the parent’s needs, a consensus meeting of experts in pediatric care and research, identifying relevant domains, and finally specific questionnaire items were selected or developed.

We conducted a comprehensive literature search to collect published questionnaires for assessment of pain and impairment in children and adolescents aged 12–17 years old. The search strategy was adapted from Meldgaard et al. [20] and updated in collaboration with an information specialist from the University of Southern Denmark. We searched the Ovid databases Medline and Embase. Literature published in English or Scandinavian languages was included.

Next, we employed forward and backward citation tracking to identify additional relevant articles facilitated by using Scopus. A single author (FGO) systematically reviewed articles by abstract and, if relevant, full-text articles were obtained (Appendix 2).

To align with COSMIN guidelines copyrighted questionnaires were excluded to allow for future implementation.

We met with the clinicians involved in examining children at the spine center to obtain their initial suggestions for clinically relevant domains.

Next, we invited participants who were either experts in pediatric or clinimetric research (HHL, LH, MD), senior researchers (SON, CLS, CN), or had clinical experience with pediatric patients (ARE, MBK, SHP). This selected expert group was composed to represent a wide diversity of professional expertise and knowledge in the field. Before the meeting, all participants were introduced to the findings of our systematic literature search and provided with a draft of the domains suggested in the pre-meeting with the clinicians.

All items were thematically divided into categories based on their classification in the International Classification of Functioning, Disability and Health (ICF) [24] to ensure that the MiRD-Kids would cover relevant categories. The complete and original versions of all the identified questionnaires were available at the meeting to facilitate the discussion on clinically relevant domains. During the meeting, participants were placed in groups of three to discuss the most relevant domains. All groups had access to a Padlet board [25], where note-taking, comments, and suggestions were possible. After the group work, the notes on the Padlet boards were discussed in a plenary session. Emerging domains were discussed for relevance, and agreement on the most relevant was reached through group consensus.

Items were formulated with the intention of being understandable by children aged 12–17-year, which is the age group most frequently assessed at the spine center [23]. We expected children within this age group to be cognitively and linguistically capable of completing the MiRD-Kids on their own. However, we also provided a by-proxy option of answering it with a parent/legal guardian. Each item was carefully drafted to align with the overarching thematic context and consistently worded to maintain uniform language throughout MiRD-Kids. This approach aimed at facilitating ease of comprehension and reporting for the children. The response options were chosen to reflect the question optimally. Categorical response options or open questions were chosen based on the purpose of the item.

The steering committee was comprised of FGO as project leader, CN as acting supervisor, HHL as a clinometric expert, and LH as a pediatrics expert. A-priori, it was decided that authors FGO, HHL, LH, and CN would formulate the final items to be included in the questionnaire to facilitate the process and make final wording of items feasible. However, all authors needed to provide feedback and approved the questionnaire before pilot testing. (See Appendix 3 for the complete wording of each item and questionnaires linked to each item used in the first version of MiRD-Kids).

#### Phase 2: Pilot testing

This phase focused on a preliminary assessment of MiRD-Kids through multiple rounds of pilot testing. All children between the ages of 12–17, seen in the pediatric outpatient track at the Spine Center of Southern Denmark were invited to complete MiRD-Kids in collaboration with a parent/guardian from June 1 to August 15th, 2023. The questionnaire was completed on paper. Following this, we conducted semi-structured interviews with questions covering the following domains: relevance to the patient’s spinal pain, comprehensiveness, and comprehensibility of the items [26]. The domains were covered by ensuring that the questions focused on the study’s aim and participants' experience (relevance), exploring all potentially relevant aspects, both general and specific (comprehensiveness), and explicitly asking about the participants' understanding of the questions and if they were clearly stated (comprehensibility).

During the interviews, children could elaborate on their answers to the individual items and express whether they felt anything was redundant, missing or repeated. The items were iteratively modified based on the answers from the prior interviews until no further issues emerged. FGO, HHL, LH, and CN discussed the completed modified MiRD-Kids with notes from the interviews and a comparison of misunderstandings and interpretations of specific questions.

This was repeated as an iterative process until it was deemed that there was no further need for modifications. The semi-structured interviews were thematically analyzed to identify recurring themes related to item clarity, relevance, and comprehensiveness.

#### Questionnaire structure

The final MiRD-Kids version was planned to be structured in two parts: one initial intended for the parent/legal guardian and one for the child. Items presented to both parents and children addressed the relevant domains as agreed upon in phase 1.

#### Phase 3: Implementation

The third phase was the implementation of the MiRD-Kids into a clinical registry at the spine center. FGO developed a protocol and an introduction video for a completed MiRD-Kids, and the clinicians were guided in the interpretation of answers at the weekly pediatric team meetings. Furthermore, FGO functioned as a contact person to aid clinicians in interpreting MiRD-Kids until they felt comfortable, and the pediatric team had the opportunity to voice concerns or suggestions at the weekly meetings, in which the implementation process was continuously evaluated. The questionnaire was set up on a digital platform that can be completed by the patient and parent/legal guardian before the assessment or completed in the waiting area.

### Statistical evaluation

Descriptive statistics were used to summarize participant demographics and response patterns. All statistical analyses were conducted in R (version 4.3).

## Results

### Phase 1: Consensus and item formulation

Five questionnaires were located from the systematic literature search: Functional Disability Inventory (FDI) [27, 28], KIDSCREEN-52 [29], Young Spine Questionnaire (YSQ) [30], Young Disability Questionnaire (YDQ) [19], and the Bath Adolescent Pain Questionnaire (BAPQ) [31]. From forward and backward citation tracking, two further questionnaires were included: Child Activity Limitations Interviews (CALI-9 and CALI-21) [32, 33].

The pre-meeting with the clinicians resulted in 38 items. It also resulted in a request for a parental part (parental-oriented questions) and two free text items (for expression of expectations in connection with the assessment). The process resulted in ten overall domains, which included 40 items for formulation, all aligned with the ICF categories.

**Fig. 2:**
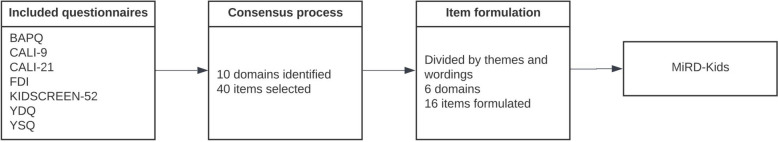
Item formulation for MiRD-Kids

Authors FGO, HHL, LH, and CN reviewed all 40 items over the ten domains. Some items had the same wording or intention, and this was used as inspiration for the formulation of the Danish items. This resulted in 16 items that were included in the first version of MiRD-Kids and tested in the first pilot test (Fig. 2). The ten domains were collapsed into six domains during the consensus process to provide a specific clinical focus.

#### Phase 2: Pilot testing

In round one, we included eight pediatric patients (aged 12 to 17) who completed MiRD-Kids in a median time of 12.5 min (range 5 to 30 min). Three children aged 16, 17, and 17, had no parental contact during completion. Some children faced challenges in understanding the question concerning medication. It was unclear to the children which medication they should report. Over-the-counter medication bought by the children themselves or by the parents/legal guardian was often not considered relevant medication. Based on this feedback from participants, we incorporated a sub-question: “Did you get it from: *GP*, *parents/legal guardian*, or *bought it/took it yourself?*” Additionally, we reorganized the order of treatment questions to improve clarity for the children.

In the school/activity domain, we removed one item due to perceived repetition and modified two other items to address comprehension difficulties encountered by the children specifically.

The changes resulting from the pilot tests can be seen in Table 1.

**Table 1:** Implemented alterations of the MiRD-Kids following the initial round of interviews

Items with issues raised during Pilot test 1	Modifications following Pilot test 1	Reasons for modification
Is one of your parents/legal guardian/siblings ill? If yes, Who is ill? If so, Would you share what illness?	Has there happened a serious life event close to you? E.g. divorce, illness or death If yes, Whom has it happened to? If yes, Describe shortly what happened	The question did not reflect all life events and illness that could have an impact on children The sub-questions were modified to fit the main question
	Addition of question: Did you get it from: GP—parents/legal guardian – bought it/took it yourself	Several children had trouble answering the medication question and did not include the pain medication that was distributed from home stock
What are your thoughts about school lately?	Are you satisfied with school?	The question was confusing and changed according to suggestions from interviewees
Do you have shorter schooldays than your classmates do?	Do you have different meeting hours compared to your classmates due to your neck- or back pain?	It did not comprehend the ways a schedule had to be changed for most of the children
Are there any specific subjects in school you don’t participate in?	Removed	Not relevant. Overlapping with other questions

**Fig. 3:**
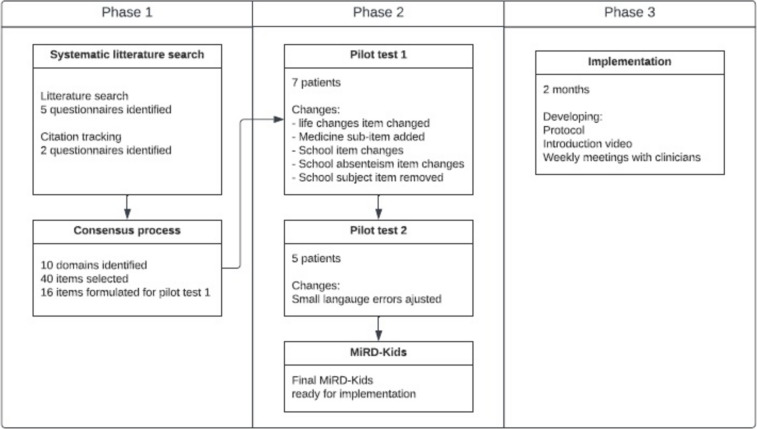
Results from the three phases used in developing of MiRD-kids

In the second round, we included another five pediatric patients (aged 12–16) who completed the modified version of MiRD-Kids, with a median time of 10 min (range 7 to 25 min). Two aged 15 and 16, had no parental contact during completion. There was no further need for changes except for minor language errors, and no further testing was carried out (Fig. 3).

#### Questionnaire structure

MiRD-Kids was structured in two parts. The first part is directed to the parent(s)/legal guardian(s) and includes the following two items:

- “Do you experience persistent or recurrent neck or back pain?”
- “Does the pain affect what you can do in your everyday life?”

The second part involves a thematic division of items and covers all six agreed-upon domains:

- Concerns: child’s own concerns from experiencing pain.
- Pain: location, duration, previous trauma, intensity, and medication consumption.
- Sleep: disturbances related to falling asleep, staying asleep, or waking up.
- Sports/activities: activity level in everyday life and in relation to sport. The domain also uncovers the ownership of the sport by the child and is socially oriented.
- Trauma: major psychological trauma.
- Treatment: previous treatment divided into health care professions.

Finally, we included two free-text items concerning expectations and any other information that the children or parent/guardian found relevant to disseminate (see Table 2 for the final version of MiRD-Kids [in English but not cross-culturally translated] and Appendix 4 for the Danish version as presented for patients).

**Table 2:** Final version of MiRD-Kids

*Question*	English	Answer
*Parental*	Are you completing this questionnaire with a parent/guardian?	Yes – No
*If yes*	Do you experience recurring neck or back pain?	Yes – No
*If yes*	Does the pain affect what you can do in your everyday life? E.g., care considerations, need for treatment, opting out of leisure activities, etc	Yes – No
*1*	Draw where you are experiencing your neck or back pain	Pain drawing
*2*	How long have you had neck or back pain?	Less than 3 months – 3–6 months – 7–12 months – More than 1 year
	Did you suffer neck or back pain after an accident? E.g., traffic accident, fall from a trampoline	Yes – No
*3*	How sore is your neck or back when you have the most pain in the last 14 days?	Scale from 0 (No pain at all) to 10 (Worst pain imaginable)
*4*	Are you taking medication for your neck or back pain?	Yes – No
*If yes*	Did you get it from: - Your doctor - Your parents/guardian - Took/purchased it yourself	Yes – No Yes – No Yes – No
	How often do you take medication for your neck or back pain?	Every day – 3–6 times a week – 1–3 times a week – Occasionally
*5*	Do you wake up at night because of neck or back pain?	Yes – Occasionally – No
	Do you have trouble falling asleep due to neck or back pain?	Yes – Occasionally – No
	Do you feel rested when you wake up in the morning?	Yes – Occasionally – No
*6*	How many hours a week do you do physical activity that makes you sweat?	Less than 1 h – 1–2 h – 3–4 h – 5–6 h – More than 6 h
	Are you doing more or less physical activity than before you had neck or back pain?	Less – Same—More
*7*	Do you avoid sports activities because of your neck or back pain? For example, soccer, gymnastics, badminton, etc	Yes – Occasionally – No
	How happy are you with your sport now?	Scale from 0 (Not happy at all) to 10 (Very happy)
	How happy were you with your sport before you got neck or back pain?	Scale from 0 (Not happy at all) to 10 (Very happy)
*8*	List three activities you find difficult to do because of your neck or back pain?	Three free text boxes
*9*	Are you avoiding activities with your friends because of your neck or back pain?	Yes – Occasionally – No
	Do you often feel lonely?	No – Yes, sometimes – Yes, often – Yes, very often
	Have you been bullied on social media or over the phone?	Don’t know – No, never – Yes, once – Yes, several times
*10*	Do you live with your parents?	Yes—No, my parents live separately—No, I live with my mother—No, I live with my father—No, I live with neither my mother nor father
*11*	Has a serious life event happened close to you? For example, divorce, serious illness or death	Yes – No
*If yes*	Whom has it happened to?	Yourself—Someone in your household—Family—Friends—Others
*If yes*	Describe briefly what happened	Free text box
*12*	Are you worried that your neck or back pain is caused by something serious?	Yes – No
*If yes*	What do you think your neck or back pain is caused by?	Free text box
	Do you think your neck or back pain will get worse or better in the future?	Much worse—Slightly worse—No change—Slightly better—Much better
*13*	How satisfied are you with your school/education?	Very dissatisfied—Dissatisfied—Neither satisfied nor dissatisfied—Satisfied—Very satisfied
	Do you have altered meeting times than your classmates because of your neck or back pain?	Yes – No
*If yes*	Which subjects are you not attending?	Free text box
*14*	Have you received treatment for your neck or back pain?	Yes – No
*If yes*	Have you seen your GP?	Yes – No
*If yes*	Did it help with your neck or back pain?	Much worse—A little worse—No change—Very little—Very much
	Have you seen a doctoral specialist (e.g. rheumatologist)?	Yes – No
*If yes*	Did it help with your neck or back pain?	Much worse—A little worse—No change—Very little—Very much
	Have you been to a chiropractor?	Yes – No
*If yes*	Did it help with your neck or back pain?	Much worse—A little worse—No change—Very little—Very much
	Have you been to a physiotherapist?	Yes – No
*If yes*	Did it help with your neck or back pain?	Much worse—A little worse—No change—Very little—Very much
	Have you been treated for your neck or back pain by others? E.g. acupuncturist, masseur or similar	Yes – No
*If yes*	Which treatment have you tried?	Free text box
*If yes*	Did it help with your neck or back pain?	Scale from 0 (Very little) to 5 (Very much)
*15*	We want to know what matters most to you when you are in pain Complete the four questions below, so that what matters most gets the highest score and what matters least gets the lowest score a.That it hurts so much b.That I can’t move around as much as I’d like c.That I can’t spend as much time with my friends d.That I get sad or angry or can’t concentrate	Scale from 0 (It matters the least) to 10 (It matters the most)
*15*	What are your expectations for your visit to the Spine Center?	Free text box
*16*	If there's something we haven't asked about that you think we should know, you can post it here	Free text box

#### *Phase 3*: Implementation

Implementing MiRD-Kids at the Spine Centre of Southern Denmark took approximately 1.5 months (from August 15th to 1st of October). The technical setup of the registry was more difficult than anticipated. In Denmark, the preferred means of communication from governmental authorities to individuals is by a common, national email-like system called ePost. This proved a challenge, as the system is designed to forward any communication to the parents/legal guardian of anyone aged 14 or younger. By contrast, children aged 15 and older have their own ePost account, but in our experience are less diligent about reading it. Overcoming these issues required some degree of day-to-day oversight and management, which was left to the secretarial staff in collaboration with FGO.

Clinicians in the pediatric team were instructed in the use of the protocol and the introductory video. The technical setup was discussed at weekly meetings, and all clinicians were familiarized with MiRD-Kids (Appendix 5, for the English protocol). The clinicians has expressed a streamlined workflow and enhanced patient presence. In the period October 2023 till March 2024, 49 (98%) has responded to the questionnaire.

#### Subsequent additions to MiRD-Kids

As a final step, the steering committee (FGO, HHL, LH, and CN) compared MiRD-Kids with the included complete pediatric questionnaires for potential meaningful additions. We recognized an opportunity to enhance MiRD-Kids by including four relevant questions from YDQ [19]. With these additions, MiRD-Kids now contains 20 items. The four items could be classified as the *concerns* domain. The YDQ have been thoroughly tested with satisfactory content validity [19].

## Discussion

### Summary of findings

We developed a clinical questionnaire – the MiRD-Kids – with items deemed relevant based on a evidence syntheses, expert consultation, consensus processes, interviews of children and their parents/legal guardians, and continuous modifications. The final version with the subsequent addition contains a main part of 20 items covering six clinically relevant domains and takes less than 30 min to complete (estimated average completion time of 10–15 min). The parental part contains three questions and can be skipped if no parent is involved. To our knowledge, this is the first standardized clinical questionnaire to assess the ICF categories of body function, activities and participation, environmental factors, and body structure for a care-seeking pediatric population with spinal pain, as research has primarily involved the prevalence of spinal pain in normal populations (e.g., school children) [30], practical consequences of general pain conditions [27, 28], or more broadly, psychological factors [29].

### Assessment of children in clinical practice

It is essential to emphasize that our clinical tool is tailored for use on children with impairments due to spine-related complaints within a hospital healthcare setting. However, we anticipate that MiRD-Kids could be applicable across various healthcare settings, given the interchangeability of the target group of children experiencing pain. Additionally, none of the 20 items across 6 domains were specific to a hospital setting. However, adaption and validation may be relevant before using MiRD-Kids in other contexts.

In a clinical setting, knowledge of a patient's needs and identifying patients at risk for poorer outcomes may lead to more patient-centered management, better use of resources, and realistic clinical treatment decisions [34]. Therefore, there is need to gather comprehensive data as accurately and timely as possible to help support the clinicians in enabling patient-centered and age-specific care for the pediatric spinal pain population. The introduction of MiRD-Kids as a patient-reported clinical questionnaire signifies an advancement in standardizing data collection of pediatric patients’ pain through the domains concerns, pain, sleep, sports/activities, trauma, and treatment.

### Clinical relevance

MiRD-Kids can play a pivotal role in preparing clinicians to address these concerns by enhancing their understanding of pain experiences and associated factors in children with spinal pain. Although MiRD-Kids is still to be tested in primary care, we see a potential for more widespread use, which could lead to more patient-centered and age-specific care. The subsequent addition of four items from the YDQ [19] does allow for future comparison between hospital and primary care sectors. Moreover, it may facilitate a more effective referral process to and within the hospital care sector if needed.

MiRD-Kids equips clinicians with a standardized instrument to complement the clinical assessment of pediatric patients aged 12–18 years, potentially empowering healthcare providers to identify and address issues, thereby enhancing patient outcomes promptly.

Lastly, MiRD-Kids may facilitate a potential systematic collection of longitudinal data for clinical and research use.

Like other musculoskeletal conditions, spinal pain in children presents unique challenges compared to other conditions. Existing tools for pediatric musculoskeletal pain generally focus on broader or non-specific pain and do not sufficiently address the specific needs of children with spinal pain, especially those seeking care in hospital settings. Furthermore, tools designed for adults do not account for developmental differences in cognition and physical function, making them not suitable for children. This highlights the need for MiRD-Kids, which is tailored to experiences and consequences of spinal pain in pediatric populations, providing clinicians with a more patient-centered and age-specific approach for assessment and treatment.

### Strengths and limitations

The methodology of combining a systematic literature search, consensus processes, and interviews strengthens the trustworthiness of the final version of MiRD-Kids. While questions from validated questionnaires were carefully integrated, we acknowledge that this does not automatically validate the MiRD-Kids in its entirety. Further validation through empirical studies with larger samples is essential before recommending its use in clinical or research settings. Specifically, comparative testing against established measures, such as the Young Spine Questionnaire, would help evaluate its clinical utility and psychometric properties.

Our systematic literature search may have missed relevant studies, as only literature published in English or Scandinavian languages was included. However, with this study's thorough content validity methodology, we believe MiRD-Kids covers the most important clinical aspects for the child-patient with spinal pain.

The involvement of patients in the development process was primarily related to the verification of our chosen items. Arguably, including patients and/or parents/legal guardians throughout all three phases could have improved MiRD-Kids further. However, considering the children’s age and our general lack of knowledge on pediatric spinal pain in general, this inclusion process would have been challenging with unclear benefits.

The children were at liberty to complete the MiRD-Kids questionnaire on their own or to involve a parent/legal guardian as they saw fit. It could be argued that either scenario is problematic: Undue involvement on part of the parent/legal guardian could influence the child's reporting, but conversely for some children a parent/legal guardian could help clarify questions and qualify answers. While each item has been validated in its original context within each separate questionnaire, our validation is primarily related to the semi-structured interviews and the strong involvement of clinicians. Building a questionnaire this way is a strength as it secures a founding in face-validity from both the patient and clinician’s perspective. Nonetheless, further psychometric validation will be required to solidify its reliability and accuracy in practice.

The question clarity was ensured through pilot testing on the target pediatric population, during which iterative modifications were made based on feedback obtained from semi-structured interviews. By the conclusion of this process, no further concerns regarding the clarity of the questions were identified. However, we acknowledge that this testing was conducted on a limited sample size. Therefore, further large-scale evaluation is necessary to establish the questionnaire’s broader applicability and comprehensibility across diverse pediatric populations, particular across different age groups.

### Perspectives

MiRD-Kids is specifically developed for children with spinal pain but has the potential for use in other musculoskeletal populations. For example, an orthopedic department could use MiRD-Kids by changing “spinal pain” to another anatomical region. Still, in that case, it should be tested for content validity and feasibility before use. Furthermore, MiRD-Kids was developed for a selected population of children with spinal pain in a hospital setting, but arguably its potential is not confined to this context. Using MiRD-Kids outside the Scandinavian context is also possible, but cultural differences should be considered during the translation process, and cross-cultural adaptations should be made accordingly [35].

## Conclusion

MiRD-Kids is the first comprehensive questionnaire for children with spinal pain seen in hospital settings and covers all ICF categories. We argue that it will prove useful for age-specific high-quality research and comprehensive clinical assessment of children aged 12 to 17 years. MiRD-Kids offers an individualized approach to pediatric spinal pain management, supporting both research and clinical endeavors.

## Supplementary Information

Supplementary Material 1.

Supplementary Material 2.

Supplementary Material 3.

Supplementary Material 4.

Supplementary Material 5.

## Data Availability

No datasets were generated or analysed during the current study.

## Abbreviations

COSMIN: Consensus-based Standards for the Selection of Health Measurement Instruments
ICF: International Classification of Functioning, Disability and Health
MiRD-Kids: MySpineData-Kids
PROM: Patient-reported outcome measures
FDI: Functional Disability Inventory
YSQ: Young Spine Questionnaire
YDQ: Young Disability Questionnaire
BAPQ: Bath Adolescent Pain Questionnaire
CALI-9 and CALI-21: Child Activity Limitations Interview
