# Supplementary material for: Development of a standardized patient-reported clinical questionnaire for children with spinal pain
Source: BMC Med Res Methodol. 2025 Jan 4;25:2. doi: 10.1186/s12874-024-02449-2 (PMC11699818; doi:10.1186/s12874-024-02449-2)
Supplement: Supplementary file 1 — Supplementary Material 1. [file 12874_2024_2449_MOESM1_ESM.docx]

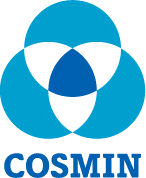
COSMIN Risk of Bias checklist

**Date:** July, 2018

**Contact**

L.B. Mokkink, PhD

VU University Medical Center

Department of Epidemiology and Biostatistics Amsterdam Public Health research institute

P.O. box 7057

1007 MB Amsterdam The Netherlands Website: [www.cosmin.nl](http://www.cosmin.nl/)

E‐mail: [w.mokkink@vumc.nl](mailto:w.mokkink@vumc.nl)

**Instructions**

*Tick* *the* *boxes* *that* *need* *to* *be* *completed* *for* *the* *article*

|  | **COSMIN** **Risk** **of** **Bias** **checklist** |
| --- | --- |
| X | Box 1. PROM development |
| X | Box 2. Content validity |
|  | Box 3. Structural validity |
|  | Box 4. Internal consistency |
|  | Box 5. Cross‐cultural validity\Measurement invariance |
|  | Box 6. Reliability |
|  | Box 7. Measurement error |
|  | Box 8. Criterion validity |
|  | Box 9. Hypotheses testing for construct validity |
|  | Box 10. Responsiveness |

| **Box** **1.** **PROM** **development** **1a.** **PROM** **design** | | | | | |
| --- | --- | --- | --- | --- | --- |
| *General* *design* *requirements* | **very** **good** | **adequate** | **doubtful** | **inadequate** | **NA** |
| 1 Is a clear description provided of the construct to be measured? | X |  |  |  |  |
| 2 Is the origin of the construct clear: was a theory, conceptual  framework or disease model used or clear rationale provided to define the construct to be measured? | X |  |  |  |  |
| 3 Is a clear description provided of the target population for which the PROM was developed? | X |  |  |  |  |
| 4 Is a clear description provided of the context of use | X |  |  |  |  |
| 5 Was the PROM development study performed in a sample representing the target population for which the PROM was developed? | X |  |  |  |  |

| *Concept* *elicitation* *(relevance* *and* *comprehensiveness)* | | **very** **good** | **adequate** | **doubtful** **inadequate** | **NA** |
| --- | --- | --- | --- | --- | --- |
| 6  7 | Was an appropriate qualitative data collection method used to identify relevant items for a new PROM?  Were skilled group moderators/interviewers used? | X  X |  |  |  |
| 8 | Were the group meetings or interviews based on an appropriate topic or interview guide? | X |  |  |  |

| 9 | Were the group meetings or interviews recorded and transcribed verbatim? |  | X |  |  |  |
| --- | --- | --- | --- | --- | --- | --- |
| 10 | Was an appropriate approach used to analyse the data? | X |  |  |  |  |
| 11 | Was at least part of the data coded independently? |  | X |  |  |  |
| 12 | Was data collection continued until saturation was reached? | X |  |  |  |  |
| 13 | For quantitative studies (surveys): was the sample size appropriate? |  |  |  |  | Not applicable |

| **1b.** **Cognitive** **interview** **study** **or** **other** **pilot** **test** | | | | | |
| --- | --- | --- | --- | --- | --- |
| 1. Was a cognitive interview study or other pilot test conducted?   *General* *design* *requirements*   1. Was the cognitive interview study or other pilot test performed in a sample representing the target population?   *Comprehensibility*   1. Were patients asked about the comprehensibility of the PROM? 2. Were all items tested in their final form? | **very** **good** | **adequate** | **doubtful** | **inadequate** | **NA** |
|  |  | | | | |
|  | YES |  | |  |  |
|  |  | | | | |
|  | X |  |  |  |  |
|  |  | | | | |
|  | YES |  |  |  |  |
|  | X |  |  |  |  |

| 18  19  20  21 | Was an appropriate qualitative method used to assess the comprehensibility of the PROM instructions, items, response options, and recall period?  Was each item tested in an appropriate number of patients?  For qualitative studies  For quantitative (survey) studies Were skilled interviewers used?  Were the interviews based on an appropriate interview guide? | X |  | |  |
| --- | --- | --- | --- | --- | --- |
|  |  | X |  |  |  |
|  |  | X |  |  |  |
|  |  | X |  |  |  |

| 22 | Were the interviews recorded and transcribed verbatim? |  | X |  |  |
| --- | --- | --- | --- | --- | --- |
| 23 | Was an appropriate approach used to analyse the data? | X |  |  |  |
| 24  25 | Were at least two researchers involved in the analysis?  Were problems regarding the comprehensibility of the PROM instructions, items, response options, and recall period appropriately addressed by adapting the PROM? | X | X |  |  |

| *Comprehensiveness* | | **very** **good** **adequate** | | **doubtful** |  | **inadequate** | **NA** |
| --- | --- | --- | --- | --- | --- | --- | --- |
| 26  27  28  29  30 | Were patients asked about the comprehensiveness of the PROM? Was the final set of items tested?  Was an appropriate method used for assessing the comprehensiveness of the PROM?  Was each item tested in an appropriate number of patients?  For qualitative studies  For quantitative (survey) studies Were skilled interviewers used? |  | | | | | |
|  |  | YES |  |  | |  | |
|  |  | X | |  | |  |  |
|  |  | X | |  | |  |  |
|  |  | X | |  | |  |  |
|  |  | X | |  | |  |  |

| 1. Were the interviews based on an appropriate interview guide? 2. Were the interviews recorded and transcribed verbatim? 3. Was an appropriate approach used to analyse the data? 4. Were at least two researchers involved in the analysis? | X    X  X  X |  |  |
| --- | --- | --- | --- |
|  |  |  |  |

| 35 | Were problems regarding the comprehensiveness of the PROM | X |  |  |  |  |
| --- | --- | --- | --- | --- | --- | --- |
|  | appropriately addressed by adapting the PROM? |  |  |  |  |  |
|  |  |  |  |  |  |  |
|  |  |  |  |  |  |  |
|  |  |  |  |  |  |  |
|  |  |  |  |  |  |  |
|  |  |  |  |  |  |  |
|  |  |  |  |  |  |  |
|  |  |  |  |  |  |  |

| **Box** **2.** **Content** **validity**  **2a.** **Asking** **patients** **about** **relevance** | | | | | | |
| --- | --- | --- | --- | --- | --- | --- |
| *Design* *requirements* | | **very** **good** | **adequate** | **doubtful** | **inadequate** | **NA** |
| 1 | Was an appropriate method used to ask patients whether each item is relevant for their experience with the condition?  Was each item tested in an appropriate number of patients?  For qualitative studies  For quantitative (survey) studies  Were skilled group moderators/interviewers used?  Were the group meetings or interviews based on an appropriate topic or interview guide? | X |  |  |  |  |
| 2 |  |  |  |  |  |  |
|  |  |  | X |  |  |  |
| 3 |  | X |  |  |  |  |
| 4 |  | X |  |  |  |  |

| 5 Were the group meetings or interviews recorded and transcribed verbatim? |  | X |  |  |  |
| --- | --- | --- | --- | --- | --- |
| *Analyses* |  |  |  |  |  |
| 6 Was an appropriate approach used to analyse the data? | X |  |  |  |  |
| 7 Were at least two researchers involved in the analysis? |  | X |  |  |  |

| **2b** **Asking** **patients** **about** **comprehensiveness** | | | | | | |
| --- | --- | --- | --- | --- | --- | --- |
| *Design* *requirements* | | **very** **good** | **adequate** | **doubtful** | **inadequate** | **NA** |
| 8 | Was an appropriate method used for assessing the comprehensiveness of the PROM?  Was each item tested in an appropriate number of patients?  For qualitative studies  For quantitative (survey) studies  Were skilled group moderators/interviewers used?  Were the group meetings or interviews based on an appropriate topic or interview guide? | X |  |  |  |  |
| 9 |  |  |  |  |  |  |
|  |  |  | X |  |  |  |
| 10 |  | X |  |  |  |  |
| 11 |  | X |  |  |  |  |

| 12 Were the group meetings or interviews recorded and transcribed verbatim? |  | X |  |  |  |
| --- | --- | --- | --- | --- | --- |
| *Analyses* |  |  |  |  |  |
| 13 Was an appropriate approach used to analyse the data? | X |  |  |  |  |
| 14 Were at least two researchers involved in the analysis? | X |  |  |  |  |

| **2c** **Asking** **patients** **about** **comprehensibility** | | | | | | |
| --- | --- | --- | --- | --- | --- | --- |
| *Design* *requirements* | | **very** **good** | **adequate** | **doubtful** **inadequate** | | **NA** |
| 15 | Was an appropriate qualitative method used for assessing the comprehensibility of the PROM instructions, items, response options, and recall period? | X |  |  | |  |
| 16 | Was each item tested in an appropriate number of patients?  For qualitative studies  For quantitative (survey) studies |  | X |  |  |  |
| 17 | Were skilled group moderators/interviewers used? | X |  |  |  |  |

| 18 | Were the group meetings or interviews based on an appropriate | X |  |  |  |  |
| --- | --- | --- | --- | --- | --- | --- |
|  | topic or interview guide? |  |  |  |  |  |
|  |  |  |  |  |  |  |
|  |  |  |  |  |  |  |
|  |  |  |  |  |  |  |
|  |  |  |  |  |  |  |
| 19 | Were the group meetings or interviews recorded and transcribed |  | X |  |  |  |
|  | verbatim? |  |  |  |  |  |
|  |  |  |  |  |  |  |
| *Analyses* | |  |  |  |  |  |
| 20 | Was an appropriate approach used to analyse the data? | X |  |  |  |  |
|  |  |  |  |  |  |  |
|  |  |  |  |  |  |  |
|  |  |  |  |  |  |  |
|  |  |  |  |  |  |  |
| 21 | Were at least two researchers involved in the analysis? |  | X |  |  |  |
|  |  |  |  |  |  |  |
|  |  |  |  |  |  |  |
|  |  |  |  |  |  |  |
|  |  |  |  |  |  |  |
|  |  |  |  |  |  |  |

| **2d.** **Asking** **professionals** **about** **relevance** | | | | |
| --- | --- | --- | --- | --- |
| *Design* *requirements* | **very** **good** | **adequate** | **doubtful** **inadequate** | **NA** |
| 1. Was an appropriate method used to ask professionals whether each item is relevant for the construct of interest? 2. Were professionals from all relevant disciplines included? 3. Was each item tested in an appropriate number of professionals? For qualitative studies   For quantitative (survey) studies  *Analyses*   1. Was an appropriate approach used to analyse the data? | X |  |  | |
|  | X |  |  | |
|  |  | X |  | |
|  | X |  |  | |

| 26 Were at least two researchers involved in the analysis? | X |  |
| --- | --- | --- |

| **2e.** **Asking** **professionals** **about** **comprehensiveness** | | | | |
| --- | --- | --- | --- | --- |
| *Design* *requirement* | **very** **good** | **adequate** | **doubtful** **inadequate** | **NA** |
| 1. Was an appropriate method used for assessing the comprehensiveness of the PROM? 2. Were professionals from all relevant disciplines included? 3. Was each item tested in an appropriate number of professionals? For qualitative studies   For quantitative (survey) studies  *Analyses*   1. Was an appropriate approach used to analyse the data? | X |  |  | |
|  | X |  |  | |
|  |  | X |  | |
|  | X |  |  | |

| 31 Were at least two researchers involved in the analysis? | X |  |
| --- | --- | --- |
