# Supplementary material for: Development of a standardized patient-reported clinical questionnaire for children with spinal pain
Source: BMC Med Res Methodol. 2025 Jan 4;25:2. doi: 10.1186/s12874-024-02449-2 (PMC11699818; doi:10.1186/s12874-024-02449-2)
Supplement: Supplementary file 2 — Supplementary Material 2. [file 12874_2024_2449_MOESM2_ESM.docx]

# Search string

## MEDLINE

MEDLINE: Function group

| 1 | (questionnaires or questionnaire or Surveys or Survey).mp. [mp=title, abstract, original title, name of substance word, subject heading word, floating sub-heading word, keyword heading word, protocol supplementary concept word, rare disease supplementary concept word, unique identifier, synonyms] |
| --- | --- |
| 2 | (function or impairment or disability or functional disability or functional limitation).mp. [mp=title, abstract, original title, name of substance word, subject heading word, floating sub-heading word, keyword heading word, protocol supplementary concept word, rare disease supplementary concept word, unique identifier, synonyms] |
| 3 | Mobility Limitation/ |
| 4 | 2 or 3 |
| 5 | (young people or school children or pediatr* or juvenile or teenager or youth or adolescen* or child or children).mp. [mp=title, abstract, original title, name of substance word, subject heading word, floating sub-heading word, keyword heading word, protocol supplementary concept word, rare disease supplementary concept word, unique identifier, synonyms] |
| 6 | Adolescent/ or Child/ |
| 7 | 5 or 6 |
| 8 | (musculoskeletal pain or musculoskeletal diseas* or musculoskeletal disorder or musculoskeletal complaint or back pain or neck pain or extremity pain).mp. [mp=title, abstract, original title, name of substance word, subject heading word, floating subheading word, keyword heading word, protocol supplementary concept word, rare disease supplementary concept word, unique identifier, synonyms] |
| 9 | Musculoskeletal Pain/ or Musculoskeletal Diseases/ or Back Pain/ or Neck Pain/ |
| 10 | 8 or 9 |
| 11 | 1 and 4 and 7 and 10 |
| **Hits** | 1457 |

MEDLINE: Disability group

| 1 | (questionnaires or questionnaire or Surveys or Survey).mp. [mp=title, abstract, original title, name of substance word, subject heading word, floating sub-heading word, keyword heading word, protocol supplementary concept word, rare disease supplementary concept word, unique identifier, synonyms] |
| --- | --- |
| 2 | (function or impairment or disability or functional disability or functional limitation).mp. [mp=title, abstract, original title, name of substance word, subject heading word, floating sub-heading word, keyword heading word, protocol supplementary concept word, rare disease supplementary concept word, unique identifier, synonyms] |
| 3 | Mobility Limitation/ |
| 4 | 2 or 3 |
| 5 | (young people or school children or pediatr* or juvenile or teenager or youth or adolescen* or child or children).mp. [mp=title, abstract, original title, name of substance word, subject heading word, floating sub-heading word, keyword heading word, protocol supplementary concept word, rare disease supplementary concept word, unique identifier, synonyms] |
| 6 | Adolescent/ or Child/ |
| 7 | 5 or 6 |
| 8 | (musculoskeletal pain or musculoskeletal diseas* or musculoskeletal disorder or musculoskeletal complaint or back pain or neck pain or extremity pain).mp. [mp=title, abstract, original title, name of substance word, subject heading word, floating subheading word, keyword heading word, protocol supplementary concept word, rare disease supplementary concept word, unique identifier, synonyms] |
| 9 | Musculoskeletal Pain/ or Musculoskeletal Diseases/ or Back Pain/ or Neck Pain/ |
| 10 | 8 or 9 |
| 11 | (intellectual disability or developmental disabilit* or disabled children or disabled persons or neurological deficit* or cerebral palsy or developmental deficit*).mp. [mp=title, abstract, original title, name of substance word, subject heading word, floating subheading word, keyword heading word, protocol supplementary concept word, rare disease supplementary concept word, unique identifier, synonyms] |
| 12 | Intellectual Disability/ or Developmental Disabilities/ or Disabled Children/ or Disabled Persons/ or Cerebral Palsy/ |
| 13 | 11 or 12 |
| 14 | 1 and 4 and 7 and 10 and 13 |
| **Hits** | 141 |

## Embase

Embase: Function group

| 1 | (questionnaire or health survey or health survey*).mp. [mp=title, abstract, heading word, drug trade name, original title, device manufacturer, drug manufacturer, device trade name, keyword, floating subheading word, candidate term word] |
| --- | --- |
| 2 | questionnaire/ or Health survey/ |
| 3 | 1 or 2 |
| 4 | (musculoskeletal function or disability or functional disability or functional limitation or impairment).mp. [mp=title, abstract, heading word, drug trade name, original title, device manufacturer, drug manufacturer, device trade name, keyword, floating subheading word, candidate term word] |
| 5 | exp musculoskeletal function/ or disability/ |
| 6 | 4 or 5 |
| 7 | (musculoskeletal pain or musculoskeletal disease or back pain or neck pain or musculoskeletal disorder or musculoskeletal complaint or extremity pain).mp. [mp=title, abstract, heading word, drug trade name, original title, device manufacturer, drug manufacturer, device trade name, keyword, floating subheading word, candidate term word] |
| 8 | exp musculoskeletal pain/ or musculoskeletal disease/ or exp backache/ or neck pain/ |
| 9 | 7 or 8 |
| 10 | (pediatrics or juvenile or adolescence or child or childhood or children or young people or school children or school child).mp. [mp=title, abstract, heading word, drug trade name, original title, device manufacturer, drug manufacturer, device trade name, keyword, floating subheading word, candidate term word] |
| 11 | Child/ or Adolescent/ or Paediatrics/ or Juvenile/ or Childhood/ or School child/ |
| 12 | 10 or 11 |
| 13 | 3 and 6 and 9 and 12 |
| **Hits** | 1410 |

Embase: Disability group

| 1 | (questionnaire or health survey or health survey*).mp. [mp=title, abstract, heading word, drug trade name, original title, device manufacturer, drug manufacturer, device trade name, keyword, floating subheading word, candidate term word] |
| --- | --- |
| 2 | questionnaire/ or Health survey/ |
| 3 | 1 or 2 |
| 4 | (musculoskeletal function or disability or functional disability or functional limitation or impairment).mp. [mp=title, abstract, heading word, drug trade name, original title, device manufacturer, drug manufacturer, device trade name, keyword, floating subheading word, candidate term word] |
| 5 | exp musculoskeletal function/ or disability/ |
| 6 | 4 or 5 |
| 7 | (musculoskeletal pain or musculoskeletal disease or back pain or neck pain or musculoskeletal disorder or musculoskeletal complaint or extremity pain).mp. [mp=title, abstract, heading word, drug trade name, original title, device manufacturer, drug manufacturer, device trade name, keyword, floating subheading word, candidate term word] |
| 8 | exp musculoskeletal pain/ or musculoskeletal disease/ or exp backache/ or neck pain/ |
| 9 | 7 or 8 |
| 10 | (pediatrics or juvenile or adolescence or child or childhood or children or young people or school children or school child).mp. [mp=title, abstract, heading word, drug trade name, original title, device manufacturer, drug manufacturer, device trade name, keyword, floating subheading word, candidate term word] |
| 11 | Child/ or Adolescent/ or Paediatrics/ or Juvenile/ or Childhood/ or School child/ |
| 12 | 10 or 11 |
| 13 | (disabled person or handicapped child or intellectual impairment or developmental disorder or neurological deficit or disabled child).mp. [mp=title, abstract, heading word, drug trade name, original title, device manufacturer, drug manufacturer, device trade name, keyword, floating subheading word, candidate term word] |
| 14 | Handicapped child/ or Intellectual impairment/ or Developmental disorder/ or Neurologic disease/ or Cerebral palsy/ |
| 15 | 13 or 14 |
| 16 | 3 and 6 and 9 and 12 and 15 |
| **Hits** | 112 |

All hits was importet til Zotero and duplicates was deleted.
