# Supplementary material for: Development of a standardized patient-reported clinical questionnaire for children with spinal pain
Source: BMC Med Res Methodol. 2025 Jan 4;25:2. doi: 10.1186/s12874-024-02449-2 (PMC11699818; doi:10.1186/s12874-024-02449-2)
Supplement: Supplementary file 3 — Supplementary Material 3. [file 12874_2024_2449_MOESM3_ESM.docx]

Detailed presentation of MiRD-Kids items and response options

| Theme | Item formulated for MiRD-Kids | Respons option for MiRD-Kids | Original questionnaire and item for item formulated for MiRD-Kids | Respons option in original question |
| --- | --- | --- | --- | --- |
| Parental part | Are you completing this questionnaire with a parent/guardian? | Yes – No | None |  |
|  | Do you experience recurring neck or back pain? | Yes – No | YSQ   - Has your father or stepfather ever had back or neck pain? - Has your mother or stepmother ever had back or neck pain? | Yes – No |
|  | Does the pain affect what you can do in your everyday life? E.g. care considerations, need for treatment, opting out of leisure activities, etc. | Yes – No | YSQ   - If he has, has it kept him home from work? - If she has, has it kept her home from work? | Often – Once in a while – Never |
| Pain | Draw where you are experiencing your neck or back pain | Drawing | MiRD   - Draw where you are experiencing your neck or back pain | Drawing |
|  | How long have you had neck or back pain? | Less than 3 months – 3-6 months – 7-12 months – More than 1 year | YSQ   - How often have you had pain in the neck? - How often have you had pain in the middle of the back? - How often have you had pain in the lower back? | Often – Once in a while – Once or twice - Never |
|  | Did you suffer neck or back pain after an accident? E.g. traffic accident, fall from a trampoline | Yes – No | MiRD   - Did the onset of the pain follow physical trauma? | Yes - No |
|  | How sore is your neck or back when you have the most pain in the last 14 days? | Scale from 0 (No pain at all) to 10 (Worst pain imaginable) | MiRD   - How bothersome has your back pain been over the last 2 weeks? | Not at all – Mildly – Moderately – Severely - Extremely |
|  | Are you taking medication for your neck or back pain? | Yes – No | MiRD   - Do you take morphine medicine for your pain? | Yes – No |
|  | If yes, Did you get it from:   - Your doctor - Your parents/guardian - Took/purchased it yourself | Yes – No  Yes – No  Yes – No | None |  |
|  | How often do you take medication for your neck or back pain? | Every day – 3-6 times a week – 1-3 times a week - Occasionally | None |  |
| Sleep | Do you wake up at night because of neck or back pain? | Yes – Occasionally – No | YDQ   - When I’m in pain, I don’t sleep so well   CALI-9   - Rate how difficult or bothersome you were doing SLEEP | Never – Once or twice – Sometimes – Often – Most of the time  Not very difficult – A little difficult – Somewhat difficult – Very difficult – Extremely difficult |
|  | Do you have trouble falling asleep due to neck or back pain? | Yes – Occasionally – No | FDI   - Getting to sleep at night and staying asleep (difficulty due to physical health) | No trouble – A little trouble – Some trouble – A lot of trouble – Impossible |
|  | Do you feel rested when you wake up in the morning? | Yes – Occasionally – No | None |  |
| Sports and activities | How many hours a week do you do physical activity that makes you sweat? | Less than 1 hour – 1-2 hours – 3-4 hours – 5-6 hours – More than 6 hours | BAPQ   - I do physical, recreational or fun activities | Never – Hardly ever – Sometimes – Often - Always |
|  | Are you doing more or less physical activity than before you had neck or back pain? | Less – Same – More | FDI   - Doing the activities in gym class (or playing sports) (difficulty due to physical health) | No trouble – A little trouble – Some trouble – A lot of trouble – Impossible |
|  | Do you avoid sports activities because of your neck or back pain? For example, soccer, gymnastics, badminton, etc. | Yes – Occasionally – No | YSQ   - Has neck or back pain ever stopped you from doing sports?   YDQ   - When I’m in pain, it’s difficult for me to do sports like I normally do   BAPQ   - I avoid activities that cause pain | Often – Once in a while – Once or twice – Never  Never – Once or twice – Sometimes – Often – Most of the time  Never – Hardly ever – Sometimes – Often - Always |
|  | How happy are you with your sport now? | Scale from 0 (Not happy at all) to 10 (Very happy) | None |  |
|  | How happy were you with your sport before you got neck or back pain? | Scale from 0 (Not happy at all) to 10 (Very happy) | None |  |
|  | List three activities you find difficult to do because of your neck or back pain? | Three free text boxes | None |  |
|  | Are you avoiding activities with your friends because of your neck or back pain? | Yes – Occasionally – No | YDQ   - When I’m in pain, it’s difficult for me to play as I normally can   FDI   - Doing something with a friend (for example, playing a game) (physical trouble or difficulty)   CALI-21   - Rate how difficult or bothersome you were doing PLAYING WITH FRIENDS   CALI-9   - Rate how difficult or bothersome you were doing THINGS WITH FRIENDS | Never – Once or twice – Sometimes – Often – Most of the time  No trouble – A little trouble – Some trouble – A lot of trouble – Impossible  Not very difficult – A little difficult – Somewhat difficult – Very difficult – Extremely difficult – N/A  Not very difficult – A little difficult – Very difficult – Extremely difficult |
|  | Do you often feel lonely? | No – Yes, sometimes – Yes, often – Yes, very often | KIDSCREEN-52   - Have you felt lonely?   YDQ   - When I’m in pain, I can feel more lonely than normal | Never – Seldom – Quite often – Very often – Always  Never – Once or twice – Sometimes – Often – Most of the time |
|  | Have you been bullied on social media or over the phone? | Don’t know – No, never – Yes, once – Yes, several times | None |  |
|  | How satisfied are you with your school/education? | Very dissatisfied – Dissatisfied – Neither dissatisfied nor satisfied – Satisfied – Very satisfied | KIDSCREEN-52   - Have you been happy at school? - Have you got on well at school? - Have you enjoyed going to school? | Not at all – Slightly – Moderately – Very - Extremely |
|  | Do you have altered meeting times than your classmates because of your neck or back pain? | Yes – No | FDI   - Being at school all day (difficulty due to physical health)   CALI-21   - Rate how difficult or bothersome you were doing GOING TO SCHOOL   YDQ   - When I’m in pain, I stay home from school   YSQ   - Have you stayed home from school because of neck or back pain? | No trouble – A little trouble – Some trouble – A lot of trouble – Impossible  Not very difficult – A little difficult – Somewhat difficult – Very difficult – Extremely difficult – N/A  Never – Once or twice – Sometimes – Often – Most of the time  Often – Once in a while – Once or twice - Never |
|  | If yes, Which subjects are you not attending? | Free text box | None |  |
| Trauma | Do you live with your parents? | Yes - No, my parents live separately - No, I live with my mother - No, I live with my father - No, I live with neither my mother nor father | None |  |
|  | Has a serious life event happened close to you? For example, divorce, serious illness or death | Yes - No | BAPQ   - There is conflict in my home. - There are fights between members of my family | Never – Hardly ever – Sometimes – Often – Always |
|  | If yes, Whom has it happened to? (tick the box) | Yourself – One in your household – Family – Friend - Other | None |  |
|  | If yes, Describe briefly what happened | Free text box | None |  |
| Concerns | Are you worried that your neck or back pain is caused by something serious? | Yes - No | BAPQ   - Pain scares me - I worry about my pain problem - When I think about my pain, it makes me upset   YDQ   - When I’m in pain, it scares me - When I’m in pain, it worries me | Never – Hardly ever – Sometimes – Often - Always  Never – Once or twice – Sometimes – Often – Most of the time |
|  | If yes, What do you think your neck or back pain is caused by? | Free text box | None |  |
|  | Do you think your neck or back pain will get worse or better in the future? | Much worse – Slightly worse – No change – Slightly better – Much better | BAPQ   - I feel hopeless about the future - I worry about the future | Never – Hardly ever –Sometimes – Often - Always |
| Treatment | Have you received treatment for your neck or back pain? | Yes - No | YDQ   - When I’m in pain, I get treatment (for example, from the doctor, chiropractor, or physiotherapist)     YSQ   - Have you been to a doctor, chiropractor or physiotherapist because of neck or back pain? | Never – Once or twice – Sometimes – Often – Most of the time  Often– Once in a while– Once or twice – Never |
|  | If yes, Have you seen your GP? | Yes – No | None |  |
|  | If yes,  Did it help with your neck or back pain? | Much worse – A little worse – No change – Very little – Very much | None |  |
|  | Have you seen a doctoral specialist (e.g. rheumatologist)? | Yes – No | None |  |
|  | If yes,  Did it help with your neck or back pain? | Much worse – A little worse – No change – Very little – Very much | None |  |
|  | Have you been to a chiropractor? | Yes – No | None |  |
|  | If yes,  Did it help with your neck or back pain? | Much worse – A little worse – No change – Very little – Very much | None |  |
|  | Have you been to a physiotherapist? | Yes – No | None |  |
|  | If yes,  Did it help with your neck or back pain? | Much worse – A little worse – No change – Very little – Very much | None |  |
|  | Have you been treated for your neck or back pain by others? E.g. acupuncturist, masseur or similar. | Yes – No | None |  |
|  | If yes,  Which treatment have you tried? | Free text box | None |  |
|  | Did it help with your neck or back pain? | Much worse – A little worse – No change – Very little – Very much | None |  |
| Subsequent addition | Overall question:  We want to know what matters most to you when you are in pain.  Complete the four questions below, so that what matters most gets the highest score and what matters least gets the lowest score |  | YDQ   - Directly copied | Directly copied |
|  | That it hurt so much | Scale from 0 (It doesn’t matter at all) to 10 (It matters a lot) | YDQ   - Directly copied | Directly copied |
|  | That I can’t move around as much as I’d like | Scale from 0 (It doesn’t matter at all) to 10 (It matters a lot) | YDQ   - Directly copied | Directly copied |
|  | That I can’t spend as much time with my friends | Scale from 0 (It doesn’t matter at all) to 10 (It matters a lot) | YDQ   - Directly copied | Directly copied |
|  | That I get sad or angry or can’t concetrate | Scale from 0 (It doesn’t matter at all) to 10 (It matters a lot) | YDQ   - Directly copied | Directly copied |
| Final part | What are your expectations for your visit to the Spine Center? | Free text box | MiRD   - Tell us in your own words, what expectations you have? | Free text box |
|  | If there's something we haven't asked about that you think we should know, you can post it here. | Free text box | MiRD   - If there is any additional information you think is relevant for us, please write it here | Free text box |

Legend:

List of abbreviations

- BAPQ: Back Pain Attitudes Questionnaire
- CALI-9: Child Activity Limitations Interview – 9 items
- CALI-21: Child Activity Limitations Interview – 21 items
- FDI: Functional Disability Inventory
- KIDSCREEN-52: Quality of Life Instrument for Children and Adolescents
- MiRD: Mine Rygdata (My Spine Data)
- YDQ: Young Disability Questionnaire
- YSQ: Young Spine Questionnaire
