# Supplementary material for: Development of a standardized patient-reported clinical questionnaire for children with spinal pain
Source: BMC Med Res Methodol. 2025 Jan 4;25:2. doi: 10.1186/s12874-024-02449-2 (PMC11699818; doi:10.1186/s12874-024-02449-2)
Supplement: Supplementary file 4 — Supplementary Material 4. [file 12874_2024_2449_MOESM4_ESM.docx]

**MiRD-Unge**

Cpr. nr.: ______________________________

Navn: ________________________________

1. **Udfylder du dette spørgeskema med en forælder/værge?**

| Ja | Nej |
| --- | --- |

**Hvis ja,**

**Forældre/værge skal svare på disse to spørgsmål:**

| Oplever du gentagende nakke- eller rygsmerter? | Ja | Nej |
| --- | --- | --- |
| Påvirker smerterne hvad du kan i din hverdag? Fx skånehensyn, behov for behandling, fravalg af fritidsaktiviteter e.lign. | Ja | Nej |

**Resten udfyldes af barn/ung**

1. Smertetegning:

Tegn hvor du oplever dine nakke- eller rygsmerter


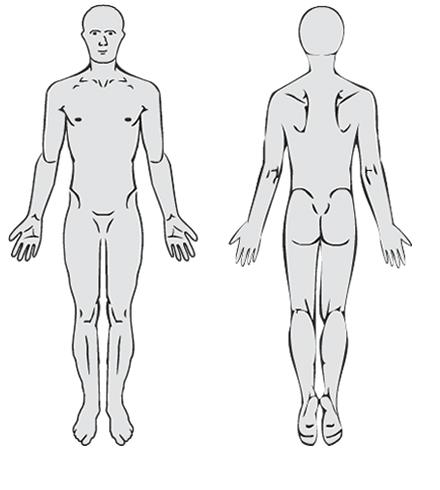


**Hvor længe har du haft nakke- eller rygsmerter?**

| Mindre end 3 måneder | 3-6 måneder | 7-12 måneder | Mere end 1 år |
| --- | --- | --- | --- |
|  |  |  |  |

**Fik du nakke- eller rygsmerter efter et uheld? Fx trafikuheld, fald fra trampolin.**

| Ja | Nej |
| --- | --- |

**Hvor ondt har du i nakke eller ryg, når du har mest ondt inden for de sidste 14 dage?**

| 0=Slet ingen smerter | | | | | 10=Værst tænkelige smerter | | | | | |
| --- | --- | --- | --- | --- | --- | --- | --- | --- | --- | --- |
| 0 | 1 | 2 | 3 | 4 | 5 | 6 | 7 | 8 | 9 | 10 |
|  |  |  |  |  |  |  |  |  |  |  |

**Får du medicin for dine nakke- eller rygsmerter?**

| Ja | Nej |
| --- | --- |

**Hvis ja,**

**Fik du det fra:**

| Lægen | Ja | Nej |
| --- | --- | --- |
| Dine forældre/værger | Ja | Nej |
| Tog/købte du det selv | Ja | Nej |

**Hvor ofte får du medicin for dine nakke- eller rygsmerter?**

| Hver dag | 3-6 gange om ugen | 1-3 gange om ugen | En gang i mellem |
| --- | --- | --- | --- |
|  |  |  |  |

**Vågner du om natten på grund af nakke- eller rygsmerter?**

| Ja | En gang imellem | Nej |
| --- | --- | --- |

**Har du svært ved at falde i søvn på grund af nakke- eller rygsmerter?**

| Ja | En gang imellem | Nej |
| --- | --- | --- |

**Føler du dig udhvilet når du vågner om morgenen?**

| Ja | En gang imellem | Nej |
| --- | --- | --- |

**Hvor mange timer om ugen laver du fysisk aktivitet, der giver dig sved på panden?**

| Mindre end 1 time | 1-2 timer | 3-4 timer | 5-6 timer | Mere end 6 timer |
| --- | --- | --- | --- | --- |
|  |  |  |  |  |

**Laver du mere eller mindre fysisk aktivitet, end før du fik nakke- eller rygsmerter?**

| Mindre | Det samme | Mere |
| --- | --- | --- |

**Fravælger du sportsaktiviteter, på grund af dine nakke- eller rygsmerter? Fx fodbold, gymnastik, badminton eller lign.**

| Ja | En gang imellem | Nej |
| --- | --- | --- |

**Hvor glad er du for din sport nu?**

| 0=Slet ikke glad | | | | | 10=Meget glad | | | | | |
| --- | --- | --- | --- | --- | --- | --- | --- | --- | --- | --- |
| 0 | 1 | 2 | 3 | 4 | 5 | 6 | 7 | 8 | 9 | 10 |
|  |  |  |  |  |  |  |  |  |  |  |

**Hvor glad var du for din sport inden du fik nakke- eller rygsmerter?**

| 0=Slet ikke glad | | | | | 10=Meget glad | | | | | |
| --- | --- | --- | --- | --- | --- | --- | --- | --- | --- | --- |
| 0 | 1 | 2 | 3 | 4 | 5 | 6 | 7 | 8 | 9 | 10 |
|  |  |  |  |  |  |  |  |  |  |  |

**Skriv tre aktiviteter du har svært ved at udføre på grund af dine nakke- eller rygsmerter?**

| 1. |
| --- |
| 2. |
| 3. |

**Fravælger du aktiviteter med dine venner på grund af dine nakke- eller rygsmerter?**

| Ja | En gang imellem | Nej |
| --- | --- | --- |

**Føler du dig ofte ensom?**

| Nej | Ja, af og til | Ja, ofte | Ja, meget ofte |
| --- | --- | --- | --- |

**Er du blevet mobbet på de sociale medier eller via telefon?**

| Ved ikke | Nej, aldrig | Ja, en gang | Ja, flere gange |
| --- | --- | --- | --- |

**Bor du sammen med dine forældre?**

| Ja | Nej, mine forældre bor hver for sig | Nej, jeg bor med min mor | Nej, jeg bor med min far | Nej, jeg bor hverken sammen med min mor eller far |
| --- | --- | --- | --- | --- |
|  |  |  |  |  |

**Er der sket en alvorlig livsbegivenhed tæt på dig? Fx skilsmisse, alvorlig sygdom eller dødsfald**

| Ja | Nej |
| --- | --- |

**Hvis ja,**

**Hvem er det sket for? (sæt kryds)**

| Dig selv | En i din husstand | Familie | Venner | Andre |
| --- | --- | --- | --- | --- |
|  |  |  |  |  |

**Hvis ja,**

**Beskriv kort hvad der er sket**

|  |
| --- |
|  |
|  |

**Er du bange for, at dine nakke- eller rygsmerter skyldes noget alvorligt?**

| Ja | Nej |
| --- | --- |

**Hvis ja,**

**Hvad tror du dine nakke- eller rygsmerter skyldes?**

|  |
| --- |
|  |
|  |

- 1. **Tror du dine nakke- eller rygsmerter bliver værre eller bedre i fremtiden?**

| Meget værre | Lidt værre | Uændret | Lidt bedre | Meget bedre |
| --- | --- | --- | --- | --- |
|  |  |  |  |  |

**Hvor tilfreds er du med din skole/uddannelse?**

| Meget utilfreds | Utilfreds | Hverken tilfreds eller utilfreds | Tilfreds | Meget tilfreds |
| --- | --- | --- | --- | --- |
|  |  |  |  |  |

**Har du ændrede mødetider i forhold til dine klassekammerater på grund af dine nakke- eller rygsmerter?**

| Ja | Nej |
| --- | --- |

Hvis ja,

Hvilke fag deltager du ikke i?

|  |
| --- |
|  |
|  |

**Har du fået behandling for dine nakke- eller rygsmerter?**

| Ja | Nej |
| --- | --- |

**Hvis ja,**

- 1. **Har du været hos egen læge?**

| Ja | Nej |
| --- | --- |

**Hvis ja,**

- - 1. **Hjalp det på dine nakke- eller rygsmerter?**

| 1= Meget værre | 2 = Lidt værre | 3 = Ikke ændret | 4 = Meget lidt | 5= Rigtig meget |
| --- | --- | --- | --- | --- |
|  |  |  |  |  |

- 1. **Har du været hos speciallæge (fx gigtlæge)?**

| Ja | Nej |
| --- | --- |

**Hvis ja,**

- - 1. **Hjalp det på dine nakke- eller rygsmerter?**

| 1= Meget værre | 2 = Lidt værre | 3 = Ikke ændret | 4 = Meget lidt | 5= Rigtig meget |
| --- | --- | --- | --- | --- |
|  |  |  |  |  |

- 1. **Har du været hos kiropraktor?**

| Ja | Nej |
| --- | --- |

**Hvis ja,**

- - 1. **Hjalp det på dine nakke- eller rygsmerter?**

| 1= Meget værre | 2 = Lidt værre | 3 = Ikke ændret | 4 = Meget lidt | 5= Rigtig meget |
| --- | --- | --- | --- | --- |
|  |  |  |  |  |

- 1. **Har du været hos fysioterapeut?**

| Ja | Nej |
| --- | --- |

**Hvis ja,**

- - 1. **Hjalp det på dine nakke- eller rygsmerter?**

| 1= Meget værre | 2 = Lidt værre | 3 = Ikke ændret | 4 = Meget lidt | 5= Rigtig meget |
| --- | --- | --- | --- | --- |
|  |  |  |  |  |

- 1. **Har du fået behandling for dine nakke- eller rygsmerter hos andre? Fx akupunktør, massør eller lign.**

| Ja | Nej |
| --- | --- |

**Hvis ja,**

- - 1. **Hvilken behandling har du prøvet?**

|  |
| --- |

- - 1. **Hjalp det på dine nakke- eller rygsmerter?**

| 1= Meget lidt | 2 | 3 | 4 | 5= Rigtig meget |
| --- | --- | --- | --- | --- |
|  |  |  |  |  |

**Vi vil gerne vide, hvad der betyder mest for dig, når du har ondt.
Udfyld skalaerne for de fire ting nedenfor, sådan at det der betyder mest får den højeste score, og det som betyder mindst for den laveste score.**

| a. | At jeg har mange smerter | Betyder mindst | | | |  |  |  |  | Betyder mest | | |
| --- | --- | --- | --- | --- | --- | --- | --- | --- | --- | --- | --- | --- |
|  |  | 0 | 1 | 2 | 3 | 4 | 5 | 6 | 7 | 8 | 9 | 10 |
|  |  |  |  |  |  |  |  |  |  |  |  |  |

| b. | At jeg ikke kan bevæge mig så meget som jeg gerne vil | Betyder mindst | | | |  |  |  |  | Betyder mest | | |
| --- | --- | --- | --- | --- | --- | --- | --- | --- | --- | --- | --- | --- |
|  |  | 0 | 1 | 2 | 3 | 4 | 5 | 6 | 7 | 8 | 9 | 10 |
|  |  |  |  |  |  |  |  |  |  |  |  |  |

| c. | At jeg ikke kan være så meget sammen med mine venner | Betyder mindst | | | |  |  |  |  | Betyder mest | | |
| --- | --- | --- | --- | --- | --- | --- | --- | --- | --- | --- | --- | --- |
|  |  | 0 | 1 | 2 | 3 | 4 | 5 | 6 | 7 | 8 | 9 | 10 |
|  |  |  |  |  |  |  |  |  |  |  |  |  |

| d. | At jeg bliver trist eller irriteret eller ikke kan koncentrere mig | Betyder mindst | | | |  |  |  |  | Betyder mest | | |
| --- | --- | --- | --- | --- | --- | --- | --- | --- | --- | --- | --- | --- |
|  |  | 0 | 1 | 2 | 3 | 4 | 5 | 6 | 7 | 8 | 9 | 10 |
|  |  |  |  |  |  |  |  |  |  |  |  |  |

**Hvad er dine forventninger til dit besøg på Rygcenteret?**

|  |
| --- |
|  |
|  |
|  |
|  |

**Hvis der er noget vi ikke har spurgt ind til, som du synes vi skal vide, kan du skrive det her.**

|  |
| --- |
|  |
|  |
|  |
|  |
