# Supplementary material for: Development of a standardized patient-reported clinical questionnaire for children with spinal pain
Source: BMC Med Res Methodol. 2025 Jan 4;25:2. doi: 10.1186/s12874-024-02449-2 (PMC11699818; doi:10.1186/s12874-024-02449-2)
Supplement: Supplementary file 5 — Supplementary Material 5. [file 12874_2024_2449_MOESM5_ESM.docx]

MiRD-Unge is completed by patients at Rygcenter Syddanmark prior to their pre-examination. It is a clinical tool developed in collaboration with clinicians - for clinicians.

It is completed by the child/young person themselves, but they can be supported by parents/guardians of their choice. The tool will uncover the child/young person's pain and impact on everyday life in their own words.

The tool is presented to patients as a questionnaire divided into several themes. Below is an overview of each theme, followed by the collection of themes. This allows each theme to be assessed individually using this protocol.

**The tool is divided into two parts
The first part** consists of a main question with two sub-questions. If the questionnaire is answered together with the parent/guardian, the parent/guardian must answer the two sub-questions. This will reveal if there is a clear impact of pain that may have an impact on family life.

**The second part** is divided into six themes of 14 questions with two final questions about expectations and further information. Each theme is discussed as follows:

# **Pain**

**Questions 1-4** uncover the child/adolescent's pain and use of medication. The child/adolescent draws pain localization, duration, previous trauma and pain intensity. In addition, they answer whether medication is taken and from where the medication is distributed.

# **Sleep**

**Question 5** covers the child/adolescent's sleep and any disturbances. All three sub-questions must be answered and therefore provide an overview of whether the child/young person wakes up, has difficulty falling asleep or wakes up rested.
The third question, whether the child/young person is rested in the morning, must be supported by strong clinical experience due to the age group and our cultural meeting times in school and education.

# **Sports/activities**

**Questions 6-9 + 13** cover the child's/adolescent's level of activity in everyday life and association with sports. The first two main questions are directly focused on sports and ask the child/young person to reflect on ownership of their sporting activity. The last two main questions (9 + 13) are socially oriented and therefore provide an overview of the child's mental state. The focus is on bullying, social skills and school attendance.

# **Trauma**

**Questions 10-11** uncover whether there is psychological trauma in the child/young person. The first question asks about the housing situation, while the second question describes major trauma and the child/young person's perception of it.

# **Concerns**

**Question 12** uncovers the child/adolescent's concerns about the pain. Sub-question two can be compared to the parent/guardian response from part one to compare mirroring patterns in the family.

# **Treatment**

**Question 14** uncovers previously tried treatments and their effect on the overall perception and impact of pain. It asks about four specific and professionally described health groups, taking into account that the child/young person and parents/guardians may not necessarily know the difference.

Question 15 summarizes where the child is most mentally troubled in their everyday life by the pain.

**Finally**, there are questions about the child's/young person's expectations of the visit and any additions that were not covered in the previous questions.
